# Constructing social Europe through European cultural heritage

**DOI:** 10.1080/14616696.2021.1913508

**Published:** 2021-04-18

**Authors:** Viktorija L.A. Čeginskas, Sigrid Kaasik-Krogerus, Tuuli Lähdesmäki, Katja Mäkinen

**Affiliations:** a Department of Music, Art and Culture Studies, University of Jyväskylä, Jyvaskyla, Finland; b Department of Finnish, Finno-Ugrian and Scandinavian Studies, University of Helsinki, Helsinki, Finland

**Keywords:** Social Europe, European Heritage Label, cultural heritage, European Union

## Abstract

The political and economic crises of the recent decades as well as the new changes brought on by globalization and digitalization have contributed to exacerbate social inequalities and injustice and revealed different social realities in Europe. The EU increasingly deals with social issues in its cultural and heritage policy. In this article, we explore the construction of this social dimension and advance the concept of ‘social Europe’ by exploring its cultural aspect based on our analysis of a recent EU heritage action, the European Heritage Label. In this action, the narrations of the European past and the attempts to foster common cultural heritage in Europe function as building blocks to create Europe as an intertwined cultural and social entity and to socialize a new generation of European citizens. We scrutinize the European Heritage Label and its notion of heritage from two perspectives. First, we analyse how the selection reports of these heritage sites construct a notion of social Europe. Second, we examine how visitors to these sites construct social Europe in their qualitative interviews. Key elements in this construction are narratives related to various values, mobility, and diversity.

## Introduction

Despite the increasing body of literature on the European Union’s (EU) cultural and heritage policies, the scholarship still lacks theoretical discussions and empirical analyses on the social dimension of these policies. In this article, we explore the construction of the social dimension of Europe and advance the concept of ‘social Europe’ by emphasizing its cultural aspect, based on our analysis of a flagship heritage action of the EU, the European Heritage Label (EHL). The EHL was established in 2011 with an aim to ‘bring to life the European narrative and the history behind it’ (EC 2020) by selecting heritage sites according to their symbolic value for Europe’s history and integration (EP&C 2011). Here, we explore how the EHL and its value-based discourse is connected to the wider notion of social Europe. With this concept, we refer to a notion of Europe that foregrounds the creation of social well-being and solidarity between its inhabitants, based on both citizen-driven collaboration and European social policies seeking to enable and promote structural equality, justice, and welfare in Europe.

Our analysis relates to socio-political developments and changes that have affected the EU member states during the recent decades. The intertwined political and economic crises as well as the new changes brought on by globalization and digitalization have contributed to exacerbating social inequalities and injustice and revealed different social realities in Europe. The discussion about alleged winners and losers of globalization as well as economic, demographic, and technological changes has caused European societies and the EU to question whether social outcomes for their citizens are equitable, for instance people’s living and working conditions and access to education, health, and social welfare systems. The EU’s responses to these crises in the field of social policy have been found insufficient. Several academic analyses have indicated that social support has been in decline in the EU since the 2000s. The EU’ social policy focuses on labour market participation and on removing regulations and barriers to trade and competition to facilitate efficient market functioning rather than redistributive intervention to improve social protection and equality (Barbier 2012; Daly 2012; Copeland and Daly 2018; Graziano and Hartlapp 2019). The discussion between Northern and Southern European states about Eurobonds or the economic reconstruction fund to respond to the economic and social consequences of the Covid-19 pandemic is the most topical example for the lack of political unanimity that undermines the legitimacy of the Union.

EU social policy is closely connected to the economy and development of the single market (e.g. Copeland and Daly 2018; Hartlapp 2020). The first reference to the social dimension of EU policy can be found in the early years of European institutional integration. The European Social Charter (CoE 1961) ensures fundamental social and economic rights and common standards of social justice, complementing the civil and political rights guaranteed by the European Convention of Human Rights (ECHR 1950). The Revised Social Charter (CoE 1996) serves as a point of reference in EU law for guaranteeing social rights and protection in a broad range of areas – such as employment, work conditions, housing, health, education, gender equality, and social welfare – and puts a specific emphasis on the protection of vulnerable persons, such as elderly people, children, people with disabilities, migrants, and women. The European Social Charter also had a great influence on the EU Charter of Fundamental Rights (EC 2012), which was proclaimed in 2000 but became legally binding only with the Treaty of Lisbon in 2009. The charter brings together fundamental personal, civic, political, economic, and social rights into EU legislation and ensures their protection and safeguarding alongside national legislations in member states. Similarly, the more recent EU social policy documents, like the European Commission’s White Paper on the Future of Europe (EC 2017a) and European Pillar of Social Rights (EC 2017b), attempt to respond to new challenges and the transformation of contemporary European societies through changed lifestyles, new patterns of work, career and labour mobility, and demographic ageing. These changes affect other areas, such as gender equality, the protection of social rights, health and care, or the global competition for skills and innovation. Hence, in the EU policy discourse, social policy is seen to serve various objectives including increasing the significance of the EU for its citizens in order to ‘create a fairer society based on equal opportunity’ (EC 2017c: 32).

These social issues have been increasingly taken up in EU cultural policy, which aims not only to promote the cultural sector but also seeks to advance explicit and implicit objectives related to the EU integration beyond the cultural sector. In ‘A New European Agenda for Culture’, the European Commission identified the social dimension as the first of its three strategic objectives aiming at ‘harnessing the power of culture and cultural diversity for social cohesion and well-being’ (EC 2018: 2). Interconnection between European cultural and social dimensions, thus, ‘brings people together’, ‘empower[s] people’, ‘increase[s] self-confidence’, enables ‘community regeneration’, ‘improves health’ and ‘psychological well-being’ and promotes ‘opportunities for all to take part and to create’, as the Agenda envisages (EC 2018: 2–3). In this process, cultural heritage is envisioned to play an important role (EC 2018: 8). Indeed, heritage sites and other cultural heritage institutions can reflect and create a more equal and just society by including various groups in their exhibitions, by reaching out to more diverse audiences, and by engaging them in core activities from curatorial practices to decision-making. In the recent EU policy documents, polyvocal, and multiple narratives, accessibility of heritage sites and museums, multilingual communication, and participatory interaction with audiences are increasingly emphasized (CoEU 2014; EP 2015), which highlights the potential of cultural heritage to strengthen the social dimension of Europe. The EHL as a flagship EU heritage action is an important instrument available for the EU to implement these policy aims.

The EHL represents a novel type of heritage action within the EU cultural policy. Although the EU has a long history of dealing with various issues of cultural heritage, its previous initiatives and projects have mainly focused on funding heritage actors at the national and local levels without content-related EU-level coordination. One of the early funding instruments included the European Parliament’s initiative of the European Historical Monuments and Sites Fund supporting the restoration and conservation of archaeological and heritage sites from its pilot phase in 1983–1995. Since the late 1990s, the European Commission has launched several cultural programmes and cooperative initiatives that explicitly focus on preserving and promoting heritage. The earliest of them, the Community action programme in the field of cultural heritage – Raphael (1997–2000) – was the Commission’s first funding programme entirely dedicated to cultural heritage with an aim to engage archaeologists and heritage professionals in its development and implementation. During the 1990s, the EU started to cooperate more closely with other transnational actors in the field of heritage. As a result, the Council of Europe’s European Heritage Days have been organized in cooperation with the European Commission since 1999, and the Europa Nostra Awards for Cultural Heritage have been awarded in cooperation with the Commission since 2002, later renamed as the European Union Prize for Cultural Heritage, and then as the European Heritage Awards in 2018. Europeana, a European digital library, archive, and museum, was initiated by the Commission in 2005, extending the EU’s interest in heritage into timely issues such as digital heritage, digitalization of (non-digital) heritage, and open access. In the 2010s, the European Parliament set up particular spaces, a visitor centre Parlamentarium (2011) and a history museum, House of European History (2017), for exhibiting European cultural heritage and the history of the EU. In the history of EU heritage initiatives, the EHL represents a move to EU-coordinated heritage actions permeated by the political goals of the European Commission.

We begin the article by explaining cultural heritage as a social process. Next, we discuss our data and methods followed by the empirical part of the article consisting of two sections. First, we explore how the selection reports of the EHL sites (2013–2019) construct a notion of social Europe. Second, we examine how people visiting EHL sites engage with the sites’ narratives and thus participate to construct social Europe ‘from below’ (Lähdesmäki et al. 2021) based on the visitor interviews conducted at eleven EHL sites in 2017 and 2018. We finish with a discussion of the core results and conclusions.

## Heritage as a social process

The concept of heritage can be defined as an act of communication (Dicks 2000), a process of emotional and cultural engagement (Bendix 2009), and a performance and cultural practice of regulation, control, mediation, and negotiation of cultural and historical values and narratives (Smith 2006; Waterton and Smith 2009). Recent scholarship in critical heritage studies emphasizes the role of heritage in the context of various power issues. In this approach, heritage is perceived as political, open to change and struggle, and both a source and a result of social conflicts, inclusion, and exclusion (e.g. Smith 2006; Graham and Howard 2008; Harrison 2013; Lähdesmäki et al. 2019a). In this view, heritage includes dissonances regarding the stories told through it, which involve the ways the past is narratively represented and memories used in public spheres (van Huis *et al*. 2020). As Harrison (2013: 4) states it, heritage emerges first and foremost from the relationship between people, objects, places, and practices and is about the people’s ‘relationship with the present and the future’ (see also Macdonald 2013). Consequently, heritage can be framed as inherently social.

Dissonance is not only an unforeseen or sometimes unfortunate implication of a certain kind of heritage or process of remembering, but intrinsic to the very nature of heritage (Smith 2006: 82; Graham and Howard 2008: 3; Kisić 2017: 25). Defining, enhancing, and fostering any heritage creates boundaries, excludes some people while including others, and positions objects, interpretations of the past, and people in certain categories. At its best, heritage is a polyvocal communication between many actors entangled with the notion of solidarity needed for societal inclusivity, cohesion, and a sense of a shared world (Delanty 2017: 178; see also Dicks 2000; Groote and Haartsen 2008). It may enable us to expose dissonances, learn from them, and thus facilitate societal dialogue and public engagement (Harrison 2013; Kisić 2017).

The temporal aspect makes heritage an active process, oriented to both the present and future, through which realities are being constructed from the selected elements of the past (Ashworth et al. 2007; Harrison 2013). However, these choices echo the past we want to have – either as a source of pride and achievement, or as a cautionary tale of difficult times we do not want to repeat. In academic debates, heritage is often seen as being about choices, not made for accuracy, or for truth, but to tell a story about who we are and where we come from, to produce and share knowledge, and to teach moral lessons. It includes diverse layers and modes of existence, being an entangled social, spatial, temporal, discursive, narrative, performative, and embodied process. Heritage narratives do not exist in isolation but are a central aspect of social life. These aspects become visible in the EU heritage policy documents and discourses.

Postmillennial Europe has faced various political, economic, social and humanitarian crises, which have deeply impacted European societies and politics. The EU’s increased interest in European heritage narratives can be perceived as an attempt to respond to some of these crises – including its own identity crises, the rise of new nationalism, and right-wing populism in Europe. Culture and heritage serve as the EU’s political tools in this process to establish the idea of Europe based on common values, political ideas, and selected narratives of the European past upon which Europeans could build their European identity (Whitehead et al. 2019; Lähdesmäki et al. 2020).

The EU’s increased interest in culture and the development of its cultural policy have been broadly examined in academia (e.g. Shore 2000, 2006; Sassatelli 2006, 2009; Patel 2013). This research commonly approaches the EU’s cultural policy aims as entangled with an attempt to strengthen integration in Europe and to create or foster a European identity. The scholars have emphasized the symbolic nature of EU cultural policy by explaining it as ‘designed both to enlarge the scope of EU power and authority and to win the hearts and minds […] of the European citizens’ (Patel 2013: 2) and as ‘being at the same time limited in reach and scope, yet distinctively oriented to the ambitious objectives of identity-building’ (Sassatelli 2009: 47). As Shore (2000, 30) argues, ‘[c]onstructing Europe requires the creation of ‘Europeans’, not simply as an objectified category of EU passport-holders and ‘citizens’ but, more fundamentally, as a category of subjectivity’. For this goal, the EU has established normative categories such as ‘common European values’, ‘European culture’ and ‘Europeanness’ through various practices and discourses in its policies (ibid.). Shore (2006: 9) describes this new type of rationality of government with a Foucauldian term as ‘EU governmentality’. It is ‘a form of power that presupposes (and works upon) the agency of individuals’ (Shore 2000: 30), and culture and cultural heritage have a central role in how the EU uses these technologies of the self (Foucault 1982).

Indeed, cultural policy has been conceived as a normalizing power that has been used for producing subjects, creating sense of belonging and governing population since the eighteenth century (Miller 2002: 5–6). Cultural heritage, notably monuments and public sculptures, as well as museums played significant roles in the nineteenth century articulation of national identities and helped make the newly established nation-states imaginable through histories and shared national property (Macdonald 2013: 141, 165). Bennett (1998: 19–20) writes how high culture was in the latter half of the nineteenth century perceived capable ‘to so transform the inner lives of the population as to alter their forms of life and behavior’ and how in these ideas, ‘self-activating and self-regulating capacities of individuals’ were seen as crucial for achieving the objectives of governance. Similarly, museums were given the ‘governmental task’ (ibid.) to ‘civilize’ the population, and this educative tradition still lives on, although the relations between cultural heritage institutions and audiences and communities are nowadays understood in more nuanced ways.

There is a substantial body of recent scholarly literature exploring various processes and practices of heritage and remembrance in culturally diverse postmillennial Europe. In these studies, the local, regional, national, European, and global or cosmopolitan dimensions of heritage are commonly approached as intertwined and mutually producing each other, which emphasizes the plurality of heritage in Europe and, hence, the perception of both Europe and heritage as plural (e.g. Macdonald 2013; Delanty 2017; van Huis *et al*. 2019; Whitehead *et al*. 2019). The studies examining how the EU has dealt with heritage and used it for creating narratives of Europe usually focus on policy documents, archived reports, and/or interviews with EU policy officers or other transnational actors (e.g. Calligaro 2013; Lähdesmäki 2014, 2017; Niklasson 2017; Jakubowski *et al*. 2019; Mäkinen 2019; Lähdesmäki *et al*. 2019b; Zito *et al*. 2019). The social dimension of EU heritage policies and initiatives and their social implication at local and grass-roots levels remain an under-researched topic.

Through explicit and implicit heritage policy the EU seeks to position itself on the continuum of the European past in ways that makes it difficult to distinguish between Europe and the EU (Lähdesmäki 2017; Lähdesmäki et al. 2020). These policies draw on their ‘symbolic’ nature as described above: cultural heritage is expected to appeal to people’s feelings of belonging, cultural and social attachments, communality, and identity by disseminating knowledge about the European past, as well as by touching people on an emotional level (Lähdesmäki 2014, 2017; Lähdesmäki et al. 2021) and thereby conceptualizing Europe as a cultural and social space. For instance, the EHL as an official heritage action has the explicit objective to create and maintain the idea of Europe as a joint community of Europeans, based on a sense of belonging and identity narrative.

As we argue elsewhere (Lähdesmäki *et al*. 2020), the role of narratives is crucial in legitimizing and sharing the choices regarding cultural heritage that, together, aim to define, guide, and transmit cultural values to future generations. This is indicated in the EHL decision that depicts the added value of the EHL action in terms of social Europe by emphasizing ‘a clear educational dimension reaching out to citizens, especially young people’ (EP&C 2011: 2). The EHL seeks to increase ‘European citizens’ understanding of the history of Europe and the building of the Union, and of their common yet diverse cultural heritage, especially in relation to the democratic values and human rights that underpin the process of European integration’ (EP&C 2011: 3) specifically through intercultural dialogue and artistic, cultural and historical education. As the EHL monitoring report states, ‘the sites are strong symbols of peace, the rule of law, welfare and democracy’ (EC 2016: 5). Indeed, the purpose of the EHL is not to promote cultural heritage because of its historical or aesthetic significance but because of its intangible aspects, such as the values frequently listed and described as European in the EHL documents.

## Data and methods

Our data consists of two distinct parts: the EHL selection reports and the visitor interviews. Currently, 48 sites in 19 participating member states hold the Label (see Table 1), which are awarded every other year. However, at the beginning of the action, the Label was awarded in yearly selection rounds. Following preselection at the national level, a European expert panel, designated by the European Commission, European Parliament, Council, and the Committee of the Regions, evaluates the sites based on three criteria: their European significance, projects and activities to highlight their European dimension, and a work plan reflecting the organizational capacity for managing the site (EP&C 2011: 4). All the criteria emphasize communicating the European dimension of the sites to wider European audiences, in particular young people.

**Table 1.** Number of EHL applicants and designations between 2013 and 2019.

Year	Number of designations	Number of applicants	Total number of EHL sites
2013	4	9	4
2014	16	36	20
2015	9	18	29
2017	9	25	38
2019	10	19	48

Our first data set consists of the five EHL selection reports (EC 2013, 2014, 2015, 2017d, 2019) and the monitoring report (EC 2016) produced by the international expert panel and published by the European Commission. In the analysis, we focus on the sites recommended for the EHL and explore how the panel assessed applications from the candidate sites according to the three criteria.

The second data set consists of 230 semi-structured interviews conducted at eleven EHL sites with visitors from nineteen EU countries, Switzerland, Russia, and Ukraine as part of a broad research project (see Lähdesmäki *et al*. 2020). These interviews were conducted between autumn 2017 and early spring 2018 at the following EHL sites: Alcide De Gasperi House Museum (Italy); Archaeological Park Carnuntum (Austria); Camp Westerbork (The Netherlands); European District of Strasbourg (France); Franz Liszt Academy of Music (Hungary); Great Guild Hall (Estonia); Hambach Castle (Germany); Historic Gdańsk Shipyard (Poland); Mundaneum (Belgium); Robert Schuman’s House (France); and Sagres Promontory (Portugal). These sites range from reconstructed archaeological remains to archives and educational or political institutes with exhibition spaces, including popular tourist attractions and small museums. We selected these sites because of their geographical location and representation of various historical periods (see also Lähdesmäki *et al*. 2020).

The EHL was established in 2011 with the intention of developing it into a high-quality label, similar to the UNESCO World Heritage List, the UNESCO Representative List of the Intangible Cultural Heritage of Humanity, and the Council of Europe’s European Cultural Routes (see EP&C 2011: Art. 5). However, its general visibility among visitors is rather low (Lähdesmäki *et al*. 2020). With a few exceptions, interviewed visitors of EHL sites were not aware of the Label and often kept confusing it with the more familiar UNESCO label during the interviews (Čeginskas 2019).

The interview questions focused on the narratives of the site, the actions and emotions invoked by the site, and the interviewees’ notion of cultural heritage. In our analysis here, we explore how the interviewees pay attention to and raise various social issues when discussing Europe. Our joint research and analysis is guided by a qualitative and interdisciplinary approach based on content analysis and multiple, collaborative readings within our multidisciplinary research team. We scrutinize in which context social aspects are raised and how they contribute to construct notions of Europe. In this article, the selected visitor quotes serve to illustrate certain prevailing ideas and opinions among them, but we do not seek to provide a quantified or comparative overview of their answers. Rather, our detailed examination of the data aims at the ‘mindful, disciplined reading of an object with a view to deeper understanding of its meanings’ (Brummett 2010: 3), and, hence, attempts to draw out the complexities of ideas among the interviewees as regards seeing Europe as a social space.

## Social Europe in EHL selection reports

European significance is the main criterion of the EHL policy discourse. For instance, the 2019 selection report states that ‘[p]resenting the European significance of a site is paramount for an application to be successful’ (EC 2019: 16). The sites are required to contextualize their sites and narratives in ‘a wider European perspective’ (ibid.) when engaging with their various audiences.

Our analysis indicates that a notion of social Europe is both explicitly and implicitly constituted in the selection reports in relation to areas like health care, housing, employment, and poverty. For instance, the Franja Partisan Hospital in Slovenia was designated the Label in 2014 for its medical and humanitarian action during the Second World War when wounded soldiers from various countries and both sides were taken to this hidden place. The report connects the medical care given in the hospital during the war with the virtues of solidarity and companionship between multiple actors. Another example is the awarding of the Werkbund Estates in Europe 1927–1932, a transnational site that deals with the idea of social housing, comprising five towns in Germany, Poland, the Czech Republic, and Austria that were built in response to the housing shortage after the First World War. The report states that this site indicates ‘social responsibility’ and demonstrates that ‘healthy living quarters could also be eﬃcient and aﬀordable buildings’ (EC 2019: 28).

In the last two reports, sites related to employment and poverty have been designated the Label. The Bois du Cazier, awarded the EHL in 2017, is a former coal-mining site related to the working conditions in the mining industry and labour immigration to Belgium in the nineteenth century. The site also focuses on the 1956 mining disaster in which 262 people of twelve nationalities died. The EHL report points out that the disaster was followed by practical acts of solidarity – help in the rescue process and fundraising for the victims’ families – but also led to a legislative revision of industrial safety regulations in Europe. In 2019, Colonies of the Benevolence was designated as a seven sub-site ensemble that ‘was established in the nineteenth century to reduce poverty through social employment in new agricultural settlements’ (EC 2019: 23). The colonies are commended for providing access to education and employment as well as preserving societal stability. The report emphasizes that the site illustrates the ‘long evolution in the European thought concerning socially marginalized people and their scarcely recognized rights as full members of society’ (ibid.). The site’s European significance is seen as lying in ‘the history of poverty reduction and social endeavours’ (ibid.), which help construct the notion of social Europe.

In most of the site evaluations, the social dimension of heritage is constituted more implicitly by selecting various elements from the past that are assumed to have contributed to developing Europe as not just an economic organization, but a community. In order to represent Europe as a community that is close to its citizens and to foster (especially young) citizens’ belonging to it, the EHL discourse emphasizes a high moral basis in virtues and values. In its selection reports, values, such as equality, justice, liberty, freedom, and tolerance are articulated as the cornerstones of European society that citizens are supposed to know, accept and, if necessary, fight for as people have done in the past. The most recent selection report claims that ‘[c]andidate sites often bear witness to the long and difficult road to the shared values of humanism, peace, freedom and democracy, human rights and the rule of law, or the quest for knowledge, social progress and welfare’ (EC 2019: 16).

In the reports, peace is one of the most frequently mentioned virtues and values (see also Mäkinen 2019). The reports refer to peace to teach present-day European citizens moral lessons about the violent past that Europe, in the form of the EU, has supposedly managed to overcome. Sites that have built their narratives on peace and reconciliation have been awarded in all five selection rounds (see Table 2). In 2013, Camp Westerbork, which ‘supports the ‘Culture of Peace and Reconciliation’ through shared European memories’ (EC 2013: 8) was designated and the Peace Palace in The Hague was described as ‘an icon and a symbol of Peace and Justice in Europe’ (EC 2013: 5). Of the 2014 sites, peace is emphasized in the narratives of Abbey Cluny, Robert Schuman’s House, and the sites of the Peace of Westphalia, Münster, and Osnabrück. The narratives of the sites awarded in 2015 also directly and indirectly advocate peace, including the Mundaneum, the European District of Strasbourg, and First World War Eastern Front Cemetery No. 123 in Poland. In the awards of 2017 and 2019, several designated sites are associated with peace, such as Javorca Church and its cultural landscape, the Former Natzweiler concentration camp and its satellite camps, the Dohány Street Synagogue Complex, Kynžvart Chateau – Place of diplomatic meetings from the nineteenth century, ‘Zdravljica’ – the Message of the European Spring of Nations, a poem written in 1848, the Lieu de Mémoire au Chambon-sur-Lignon, and the Site of Remembrance in Łambinowice. Their common narrative is that reconciliation and peaceful co-existence have the potential to overcome the destruction of war and hostility and create space for solidarity to grow: after shared experiences of suffering, new wars can be prevented together through social justice, equality, and welfare. However, the social aspects underlining this narrative are not explicitly articulated or emphasized in the selection reports.

**Table 2.** List of EHL sites awarded between 2013 and 2019.

2013	Archaeological Park Carnuntum, **Austria**; Peace Palace, **The Netherlands**; Great Guild Hall, **Estonia**; Camp Westerbork, **The Netherlands**
2014	The Heart of Ancient Athens, **Greece**; Abbey of Cluny, **France**; Residencia de Estudiantes, **Spain**; Kaunas of 1919–1940, **Lithuania**; Archive of the Crown of Aragon, **Spain**; General Library of the University of Coimbra, **Portugal**; Franja Partisan Hospital, **Slovenia**; Union of Lublin, **Poland**; Münster and Osnabrück – Sites of the Peace of Westphalia, **Germany**; The May 3, 1791 Constitution, **Poland**; Museo Casa Alcide De Gaspari, **Italy**; Robert Schuman’s House, **France**; The Historical Gdańsk Shipyard, **Poland**; Hambach Castle, **Germany**; Pan-European Picnic Memorial Park, **Hungary**; Charter of Law of Abolition of the Death Penalty, **Portugal**
2015	Krapina Neanderthal Site, **Croatia**; Mundaneum, **Belgium**; World War I Eastern Front Cemetery No. 123, **Poland**; Olomouc Premyslid Castle and Archdiocesan Museum, **Czech Republic**; Sagres Promontory, **Portugal**; The Imperial Palace, **Austria**; European District of Strasbourg, **France**; The Historic Ensemble of the University of Tartu, **Estonia**
2017	Dohány Street Synagogue Complex, **Hungary**; Javorca Church and its cultural landscape, **Slovenia**; Leipziǵs Musical Heritage Sites, **Germany**; Former Natzweiler concentration camp and its satellite camps, *transnational site*: **France and Germany**; Sighet Memorial, **Romania**; Bois du Cazier, **Belgium**; Village of Schengen, **Luxembourg**; Maastricht Treaty, **The Netherlands**
2019	Underwater Cultural Heritage of the Azores, **Portugal**; Site of Remembrance in Łambinowice, **Poland**; Archaeological Area of Ostia Antica, **Italy**; Werkbund Estates in Europe, *transnational site*: **Austria, Czech Republic, Germany, Poland**; Lieu de Mémoire au Chambon-sur-Lignon, **France**; Living Heritage of Szentendre, **Hungary**; Colonies of Benevolence, **Belgium**; Kynžvart Chateau –Place of diplomatic meetings, **Czech Republic**; Zdravljica – the Message of the European Spring of Nations, **Slovenia**; The Three Brothers, **Latvia**

Another aspect mentioned in several evaluations is mobility and crossing borders, in terms of trade routes and economic exchange as well as the mobility and migration of people. These evaluations seek to show that despite differences between people in various parts of Europe, mobility enables encounters and interaction through which Europeans can discover what unites them. This, in turn, can facilitate the mutual solidarity needed for Europe to develop into a community with a joint social vision. Various examples of mobility across borders from the distant past in the reports include Carnuntum in the Roman Empire; Abbey Cluny, founded in 910 as an administrative centre of monastic networks in Europe ‘facilitating the circulation of people, books, artistic ideas, and scientific knowledge across national borders’ (EC 2014: 4); and the fifteenth-century Great Guild Hall connected to economic and cultural interaction within the Hanseatic League (EC 2013: 6). Exemplifying a more recent past, in 2017 the Village of Schengen was designated the EHL as a site where the Schengen Agreement was signed in 1985 and now ‘makes European integration tangible’ (EC 2017d: 17).

Similarly, diversity is frequently discussed in the EHL reports as part of the required European significance of the sites. The reports emphasize diversity in terms of multiethnicity, multilingualism and diverse religions (e.g. Carnuntum, Union of Lublin, Hambach Castle, De Gasperi House, Archaeological Area of Ostia Antica, and Living Heritage of Szentendre). The report authors highlight that Europe has always consisted of diverse groups with distinct languages, cultures, and religions, thereby explaining the present diversity of Europe with its past. With the EHL, cultural, linguistic, and religious diversity are brought under the common umbrella of ‘European cultural heritage’. The goal is to make Europeans aware of both past and present diversity and perceive it as a specific characteristic that defines Europe as an inclusive community to which members from diverse backgrounds can belong. The logic of this diversity discourse is similar to one on mobility: this sense of belonging can potentially develop into solidarity that can contribute to the notion of social Europe.

In sum, the notion of social Europe is produced in the EHL reports in multiple ways and aim to strengthen faith in the future. By explicitly mediating the social dimension of cultural heritage, together with keywords such as innovation and progress, the EHL’s aim is to show how things have improved in Europe during the past centuries. The selective use of the past in relation to social issues also helps to create a sense of continuity, highlighting that core structures of modern societies, such as educational or medical institutions, never lose their value. At the same time, the EHL rhetoric links social issues with efforts to create unity through highlighting values such as peace or cultural phenomena like mobility and by emphasizing Europe’s cultural diversity. The report authors use unity, diversity, and positive values, with strong emphasis on cooperation, as entangled aspects of the ‘social Europe’ under construction and citizens’ belonging to it.

## Social Europe in EHL visitor interviews

Social Europe is constructed in the interviews both as a contemporary reality people experience personally through mobility and cultural diversity and an (idealized) understanding of creating social justice and equality. The data analysis revealed that notions of diversity, mobility, and values that the EHL promotes as ‘European’, are integral to the visitors’ processes of ‘imagining Europe’ as a cultural and social space. The previous section showed that all three are also central to how Europe is constructed in the EHL documents. These notions are key to how the visitors understand ‘social Europe’ as grounded in their everyday experiences and practices but also in an idealization of what they expect ‘Europe’ to achieve. Their personal encounters with diversity, practices of mobility, and negotiation of values reflect and influence their interpretations and understandings of social justice, social equality, and welfare.

The visitors spoke about shared European values, notions of commonality, and solidarity between Europeans despite their cultural differences. Their value discussions implied a temporal continuity: certain values, such as democracy and human rights, were understood as ancient, typical to Europe ‘for a long time’ and at the same time topical. A major temporal rupture in this trajectory is created to distance the positively perceived present reality from the sufferings related to the Second World War. Another rupture is produced by some visitors who imagine a positive past of Europe that is threatened by recent critical developments and ‘crises’ leading to more pessimistic future imaginaries. Some of our interviewees referred to past and contemporary challenges – for instance, the rise of nationalism and the extreme right across Europe – to maintaining ‘the basic values of Europe, so human rights, democracy, the rule of law’ (VS4/17). According to a young Belgian student, ‘all those who vote for the extreme right, they don’t want to be in Europe anymore’ (VS9/10) and in this respect contribute to undermine democratic principles and liberal achievements connected to what she understood to represent Europe. Likewise, an elderly French woman spoke about her fears of how increased nationalism in several European countries today resulted in a dangerous ‘retrograde matrice’, a backwards going matrix (VS5/6).

Our data reveals that the idealized notions of Europe as a value community that guarantees principles of democracy and general freedoms ‘without consideration to social status or questioning origins’ (VS7/12) may clash with people’s readiness to share privileges and standards in their everyday life. While many interviewees supported the idea of solidarity between European countries and granting access to the same set of social and political rights to all European citizens, the data revealed also a discrepancy between the prevailing value discourse and people’s actual social behaviour and everyday practices. For example, while taxes can build social welfare and equality, a young Italian student (VS1/5) stated that he would ‘never pay my taxes for a French person’. This is another example in which the idealized value discourse clashed with selective economic solidarity in practice. Other, predominantly Western, interviewees also talked about the Eastern enlargement of the EU (2004 and 2007) as lowering bars so that ‘everybody could be part of the European Union’ (VS3/9). They often considered it a mistake to incorporate post-communist countries that still struggled with economic and political problems related to their past.

The comments indicate tensions and ruptures between strengthening social bonds between all European countries, some European countries, or only at the national level. Most of them allude to the hierarchical view that Europe’s core was primarily shaped by values and political principles represented by the EU’s Western founding members. The EU is still primarily an economic and political union, in which different interests and visions persist among its member states. Economic and political considerations underlie and shape the relationship between the EU and the member states, between European countries as well as people’s individual actions and attitudes. The value discourse alone does not overcome contradictions and conflicts within member states or lead to a stronger cohesion among European peoples.

The emphasis on a European-wide significance of cultural heritage, in terms of a shared heritage in Europe, can give a different meaning to the same value discourse and bridge various, also opposing positions. According to a Polish interviewee (VS8/20), the EHL award helped to acknowledge the relevance of the Solidarity movement beyond the Polish context and revealed its impact for Western European citizens not directly affected by the events. In turn, this affected the notion of ‘Europe with which we identify ourselves, or [with which] we want to identify: [the] free, open, democratic – [it] has shifted to the east. Therefore, it [the Solidarity movement] is important for the whole of Europe’. Value discourse and social behaviour are interrelated and the extent to which people identify with a particular heritage discourse affects their actions and attitudes. However, the way in which a value rhetoric translates in precise everyday practices and concrete social relations always needs contextualization. In the European context, however, cultural heritage operating on the basis of learning from the past and emphasizing the transnational and intercultural dimension of the ‘European’ value discourse could pave a shared understanding of seeing Europe as a (conceptual) social space.

Similarly to the authors of the EHL documents, visitors to the EHL sites saw (national and cultural) diversity as characteristic of Europe. Visitors described Europe as being different, diverse, manifold, progressive, sharing common goals, and constituting a place where no animosity existed between neighbours. Their emphasis on a ‘cohesive spirit among the nations’ (VS7/21) highlighted the EU’s relevance for preventing wars and guaranteeing civil and human rights and democratic freedoms for the people living on the continent. Their interpretation often overlapped with the EU narrative that reiterates peace and diversity as central elements of the European community in the making.

Cross-border mobility, emphasized in the EHL reports, was also a key issue in the visitor interviews and played a role for the process of imagining Europe (see also Lähdesmäki *et al*. 2021). Visitors connected mobility to the four freedoms of the EU, especially the free movement of people, and gave examples of student exchange, expatriation for work, tourism and holiday trips that enable Europeans to ‘cruise around all Europe’ (VS7/10) and visit different places as part of their everyday life and leisure time (see also Lähdesmäki *et al*. 2020). Several people referred to the Schengen Agreement as the key treaty behind their mobility, thereby referring to a temporal rupture: while mobility is a natural part of our present-day reality due to relaxed border controls, it is also very different from what used to be ‘normal’ in the past. Other interviewees emphasized the long continuity behind contemporary mobility. For instance, visitors to Carnuntum frequently drew parallels between borderless Europe in their day and the Roman Empire that spread across half of Europe. Young people, in particular, emphasized that mobility resulted in revealing communality and common ground among Europeans of different nationalities. However, they often referred to privileged experiences in the context of the ‘Erasmus club’, which is for students only. Moreover, the majority of the interviewed visitors were originally from West European countries and we noted that they often viewed East Europeans’ mobility within Europe differently. Several West European interviewees spoke about their fear that the influx of East European workers would have or already had had an impact on the labour market in their home countries. Elsewhere we have discussed how the interviewees’ age-specific experiences as well as mobility experiences affected their positions on certain issues (see Lähdesmäki *et al*. 2021). Hence, unsurprisingly, the mobility of migrants from outside Europe divided interviewees between those who endorsed their right of mobility for humanitarian reasons and those who rejected it for economic, security, and ideological reasons. While some visitors saw ‘too much’ mobility as a problem, others interpreted ‘too little’ of it negatively: the recently reinstated border controls and new fences in some EU member states, which create a sense a ‘Fortress Europe’, were seen to hamper European social cohesion. The following quote of a Belgian visitor (VS9/1) alludes to precisely these ideas and constructs an idea of a social Europe based on notions of solidarity, support and inclusion beyond citizenship and nationality:

There is a dimension of the fortress Europe that I really dislike and so, for me, it‘s sharing something with other people but that’s not limited to Europe. I don’t like the idea of identity, that it’s *Europe versus* something else. More in an inclusive way, sharing values and the idea of solidarity, so that people who are fortunate can help other people, and so on. That’s more the idea that I like. But not the closed idea of Europe.

The visitors commonly adopted and repeated EU rhetoric when conceptualizing Europe and the ‘European’ as an imagined cultural and social space. They approached Europe in terms of legal and political integration and referred to values, such as equality, social justice, or civic rights, as a central means of depicting Europe. Visitors’ imagination of Europe was strongly shaped by actual EU policies and discourses, not only with references to mobility and the Schengen zone, but also in other contexts. For example, a young Italian student (VS1/2) linked her understanding of Europe with EU policies:

To know what Europe is, where we came from and what we want to do for Europe and why we have to do something for Europe. Why we have to collaborate, why we have to take part in the European Union.

In sum, while there is a sense of cultural closeness and even solidarity between Europeans of different backgrounds, social issues and feelings of social inequality create a gap between various groups of people in Europe. The idea of a social Europe manifests itself in the visitors’ perceptions of Europe as an entity that provides peace and welfare, creates conditions that favour personal mobility, and ensures democracy and freedoms. Mobility and diversity are both understood as positive as long as they refer to the interviewees’ personal freedom. However, mobility – more than diversity and values – constructs distinct perceptions, politics, and practices of inclusion/exclusion associated with questions of who belongs to Europe and who is European. Thus, mobility is integral to the interpretation of the discourse on both values and diversity. This raises the issues of whose mobility, whose values, and whose (and ‘how much’) diversity are fundamental to the social dimension of Europe.

## Concluding discussion

Several scholars have criticized the EU for ‘marginalization and tokenization of social policy as compared with macroeconomic and financial concerns’ (Barbier 2012: 377). Although heritage experiences make it possible to raise awareness of socio-political questions, such as social in/justice, social in/equality and poverty, and to encourage visitors to act upon them through cultural heritage, our analysis of EHL selection reports and visitor interviews suggests that such links between cultural heritage and the social dimension of Europe are currently rather weak in the EHL. Nevertheless, our research indicates how discourses on European cultural heritage raise and arise from various social and social-political issues – especially through the selection of sites that focus on health care, housing, employment, and poverty. The EHL sites help to negotiate the meaning of these social issues through European historical contexts and invoke shared experiences for their visitors. Through their representations and narrations of the European past, the sites can use various technologies of the self (Foucault 1982) seeking to activate the visitors to promote the EU objectives. Our analysis shows how normative categories such as ‘common European values’, ‘European culture’ and ‘Europeanness’ are created and used in both the EHL evaluation reports and visitor interviews (Shore 2000).

Our findings contribute to recent research in critical heritage studies on the construction of the idea of Europe by extending their scope with a focus on social dimension. Our two data sets underline cultural diversity as an essential element of Europe. Even though the diversity rhetoric may sound inclusive, it is useful to acknowledge the problems inherent in the EU’s use of the notion of diversity for fostering the unity of Europe and the fact that the tension between unity and diversity is not easily reconciled (Shore 2006). Both evaluators and visitors emphasize values that lay the ground for peaceful social relations in Europe, such as solidarity, democracy, and freedom. Both groups include idealistic discourses of Europe based on imagined or desired aspects of social justice and equality in current and future European societies. These discourses are closely linked to their performativity and agency: imagining future Europe includes efforts to make it reality through educative activities at the heritage sites. For instance, the EHL evaluators sought to teach present-day European citizens lessons about the European past in order to promote a peaceful and prosperous European future. Respectively, many of the EHL visitors considered it important to learn from history at the heritage sites in order not to repeat past mistakes. However, the visitor interviews reveal social binaries – privileged versus underprivileged, mobile versus non-mobile, and West Europeans versus East Europeans – through which the interviewees constructed ‘their’ social Europe. We have discussed elsewhere, in connection with the construction of ideas about Europe and belonging to Europe, that various other personal and social locations equally determine people’s complex constructions. These include, for example, gender, social enculturation, level of education, partiality of the curricula in different European countries, habitus, or personal preferences (see Lähdesmäki *et al*. 2021: Chapter 7). Both data sets thus reflect the interpretation of the past and the construction of cultural heritage through selective remembering (Ashworth *et al*. 2007; Harrison 2013).

In both data sets, mobility and border-crossing are core themes and understood as cultural practices that help construct ideas of communality. As such, they are also linked to the notion of social Europe. However, the understanding of mobility and movement as a social phenomenon that characterizes Europe affects people’s views on who has a right to belong to it (see e.g. Favell 2008; Recchi and Favell 2009). The impact of mobility on belonging raises the question of how inclusive such a ‘social Europe’ is, who participates in it, and who can claim social rights. Both the EHL report authors and most of the interviewed visitors see mobility as increasing belonging to Europe and promoting the understanding of Europe as a social entity with equal rights through people’s personal experiences of mobility and interaction with other Europeans and their ‘cultures’. In practice, however, policies and discourses of mobility treat and affect diverse groups of citizens and non-citizens in Europe in different ways, which actually broadens social inequalities. In today’s Europe, the political cleavage between mobile and non-mobile EU citizens affects people’s attitudes to European integration, their association with the EU, and their willingness to transfer sentiments usually associated with the national to the ‘European’ (see also Bauböck 2019a; Fine 2019: 130; Kuhn 2015; Risse 2004). The context of current (im-)mobilities of migrants, refugees, and asylum seekers in Europe influences our understandings of what Europe is. This context creates a challenge for participation, social justice and rights and establishes new categories of citizenship that transcend the traditional framework of nation states (Bauböck 2005, 2007, 2019b; Wiesner *et al*. 2018, 11). Indeed, while in the EHL reports, mobility is a practice and phenomenon that characterizes Europe only in positive and inclusive ways, in the EHL visitor interviews, the mobility of ‘others’ – i.e. people moving from poorer to richer regions of Europe or from outside Europe – was sometimes seen as posing a threat to the receiving countries or Europe at large.

Today, both EU heritage policy makers and heritage practitioners in Europe are concerned to establish heritage sites and museums as places that not only display cultural items and heritage objects but enter into a dialogue with their audiences, diverse heritage communities, and society at large. If heritage sites succeed in creating polyvocal interaction between various groups and touching people emotionally, they can contribute to increasing empathy and solidarity (e.g. Delanty 2017; Kisić 2017). These are necessary preconditions for constructing a social Europe, based on acknowledging the right to the same level of welfare in all member states and the responsibility to correct structural inequality as a joint endeavour of the EU’s member states and citizens. Such a notion of Europe could lay the ground for a more active social policy and push this policy higher up the EU agenda. Hence, European cultural heritage policies and practices both can and should contribute to make Europe more ‘social’, particularly if they become increasingly citizen-driven and participatory.

## Referred interviews

VS1/2: Interview December 2017, Alcide De Gasperi House Museum, Italy. Italian, female, 18–25 years.

VS1/5: Interview December 2017, Alcide De Gasperi House Museum, Italy. Italian, male, 18–25 years.

VS3/9: Interview February 2018, Camp Westerbork, The Netherlands. Dutch, female, 61–65 years.

VS4/17: Interview January 2018, Lieu d’Europe, France. German, female, 18–25 years.

VS5/6: Interview October 2017, Franz Liszt Academy of Music, Hungary. French, female, 56–60 years.

VS7/10: Interview September 2017, Hambach Castle, Germany. German, female, 61–65 years.

VS7/12: Interview September 2017, Hambach Castle, Germany. German, female, 71–75 years.

VS7/21: Interview September 2017, Hambach Castle, Germany. German, male, 76–80 years.

VS8/20: Interview September 2017, Historic Gdánsk Shipyard, Poland. Polish, male, 36–40 years.

VS9/1: Interview January 2018, Mundaneum (Belgium). Belgian, male, 31–35 years.

VS9/10: Interview January 2018, Mundaneum (Belgium). Belgian, female, 18–25 years.
